# Supplementary material for: Ultrasound on the Frontlines: Empowering Paramedics with Lung Ultrasound for Dyspnea Diagnosis in Adults—A Pilot Study
Source: Diagnostics (Basel). 2023 Nov 9;13(22):3412. doi: 10.3390/diagnostics13223412 (PMC10670426; doi:10.3390/diagnostics13223412)
Supplement: Supplementary file 1 [file diagnostics-13-03412-s001.zip › File S1. EMSC SUPPLEMENTARY MATERIALS LUS.pdf]

WZÓR

Załącznik nr 4[41)]

KARTA MEDYCZNYCH CZYNNOŚCI RATUNKOWYCH

Oznaczenie dysponenta zespołu ratownictwa medycznego (ZRM)	Kod ZRM realizującego zlecenie:	Nr zlecenia wyjazdu:

I - WYWIAD

MIEJSCE ZDARZENIA
- w domu........................ ☐
- w miejscu publicznym ☐
- w ruchu uliczno-drog. ☐
- w pracy ☐ w szkole ☐
- w rolnictwie................ ☐

II - BADANIE

GLASGOW-COMA-SCALE

OTWIERANIE OCZU
- spontanicznie................4
- na głos...........................3
- na ból..............................2
- brak.................................1

REAKCJA SŁOWNA
- zorientowany.................5
- splątany..........................4
- niewłaściwe słowa..........3
- niezrozumiałe dźwięki....2
- brak.................................1

REAKCJA RUCHOWA
- wykonuje polecenia........6
- lokalizacja bólu..............5
- ucieczka od bólu............4
- zgięciowa........................3
- wyprostna........................2
- brak.................................1

SUMA ☐☐

RTS

CZ. ODDECHÓW
- 10-29..........4
- >29.............3
- 6-9..............2
- 1-5..............1
- brak.............0

RR SKURCZOWE
- >89..............4
- 76-89...........3
- 50-75...........2
- 1-49.............1
- 0..................0

GCS
- 13-15...........4
- 9-12.............3
- 6-8...............2
- 4-5...............1
- 3..................0

SUMA ☐☐

UKŁAD ODDECHOWY

częstość oddechów... ☐☐ /min
- duszność........ T N
- sinica............. T N
- bezdech......... T N

	L	P
szmer prawidł...	☐	☐
furczenie..........	☐	☐
świsty..............	☐	☐
trzeszczenie.....	☐	☐
rzężenie...........	☐	☐
brak szmeru.....	☐	☐
inne:................	☐	☐

Saturacja..... ☐☐ %

ŹRENICE

Reakcja na światło:	L	P
prawidłowa.......	☐	☐
powolna...........	☐	☐
brak.................	☐	☐

szerokość:	L	P
normalna..........	☐	☐
wąska..............	☐	☐
szeroka............	☐	☐

CIŚNIENIE TĘTNICZE ☐☐☐ / ☐☐☐ mm Hg

TĘTNO ☐☐☐ /min miarowe ☐ niemiar. ☐

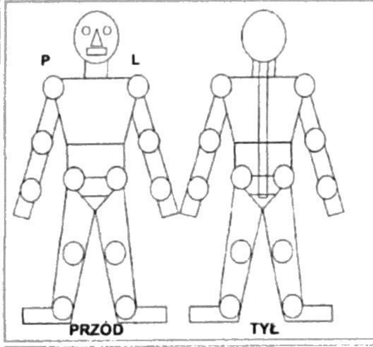

- ☐ brak obrażeń
- O Złamanie otwarte
- Z Złamanie zamknięte
- W Zwichnięcie
- S Stłuczenie
- R Rana
- K Krwotok z rany
- M Zmiażdżenie
- A Amputacja
- N Ból nieurazowy
- P Oparzenie
- ☐ stopnia ☐☐☐ %
- ☐ stopnia ☐☐☐ %
- ☐ oparzenie wziewne

Obrażenia anatomiczne kwalifikujące do centrum urazowego
- ☐ penetrujące rany głowy i tułowia lub urazy tępe z objawami uszkodzenia narządów wewnętrznych głowy, klatki piersiowej i brzucha
- ☐ amputacja kończyny powyżej kolana lub łokcia
- ☐ rozległe zmiażdżenia kończyn
- ☐ uszkodzenie rdzenia kręgowego
- ☐ złamanie kończyny z uszkodzeniem naczyń i nerwów
- ☐ złamanie co najmniej dwóch proksymalnych kości długich kończyn lub miednicy

OBJAWY
- wstrząs......... T N
- NZK.............. T N
- obj. oponowe.. T N
- drgawki.......... T N
- afazja............. T N
- wymioty.......... T N
- biegunka......... T N
- krwawienie...... T N
- obrzęki........... T N
- zasłabnięcie.... T N

INNE
- ciąża.............. T N
- poród............. T
- chor. zakaźna. T

SKÓRA

Wygląd:
- w normie..... ☐
- blada........... ☐
- rumień......... ☐
- zażółcenie... ☐
- sinica obw.... ☐
- sinica centr.. ☐

Wilgotność:
- w normie..... ☐
- wilgotna....... ☐
- sucha.......... ☐

Temperatura:
- w normie..... ☐
- chłodna....... ☐
- ciepła.......... ☐

JAMA BRZUSZNA
- w normie......... ☐
- bolesność palpacyjna...... ☐
- brak perystaltyki..... ☐
- objawy otrzewnowe.... ☐

OCENA PSYCHO-RUCH.
- w normie........ ☐
- spowolniały.... ☐
- pobudzony...... ☐
- agresywny...... ☐

TONY SERCA
- czyste/głośne......... ☐
- stłumione............... ☐
- inne:....................... ☐

NIEDOWŁAD / PORAŻENIE ☐

	L	P
kończyna górna	☐	☐
kończyna dolna	☐	☐

ZAPACH Z UST
- rozpuszczalnik org... ☐
- alkohol.................... ☐
- inne:....................... ☐

POZIOM GLUKOZY ☐ mg% ☐ g/l

EKG
- Rytm zatokowy.. ☐
- Tachykardia nadkomorowa.... ☐
- Tachykardia komorowa.......... ☐
- Migotanie/ trzepotanie przedsionków.... ☐
- AV blok............. ☐
- sVES................ ☐
- VES.................. ☐
- VF/VT.............. ☐
- Asystolia.......... ☐
- PEA.................. ☐
- Rozrusznik....... ☐
- OZW................. ☐
- Zawał:.............. ☐
- Inne:.........................

OPIS

III - ROZPOZNANIE

OPIS	KOD ICD10	KOD ICD10	KOD ICD10

IV - POSTĘPOWANIE Z PACJENTEM

CZYNNOŚCI

odsysanie........... ☐	defibrylacja.......... ☐	kołnierz................ ☐	linia żyl. obw....... ☐
went. workiem..... ☐	stymulacja zew..... ☐	deska ortoped....... ☐	linia żyl. cent....... ☐
rurka UG............. ☐	kardiowersja......... ☐	materac próż........ ☐	cewnikowanie....... ☐
intubacja............. ☐	masaż serca......... ☐	unieruchomienie. ☐	sonda żołąd......... ☐
respirator............ ☐	EKG....................... ☐	opatrunek........... ☐	monitorowanie..... ☐
tlenoter. bierna... ☐	teletransmisja...... ☐	inne..	

ZASTOSOWANE LEKI, WYROBY MED. (nazwa, dawka, droga podania)

ZALECENIA / UWAGI KIEROWNIKA ZESPOŁU

V - DANE PACJENTA I PRZEKAZANIE PACJENTA

DANE PACJENTA

Imię: ....

Nazwisko: ....

Adres zamieszkania:.......................................

ul:...nr:..........m:.........

Rodz. i nr. dok. tożsamości: ....

....

Ident. NFZ: ☐☐☐ **Data urodzenia / wiek:** ☐☐☐☐-☐☐-☐☐

NUMER PESEL pacjenta: ☐☐☐☐☐☐☐☐☐☐☐

Podpis i pieczątka kierownika ZRM: Zespół (S/P)

data udzielenia pomocy ....

Decyzja podmiotu leczniczego:

☐ Przyjęcie pacjenta ☐ Odmowa przyjęcia

pieczęć IP/SOR podpis i pieczątka lekarza

Przekazanie pacjenta w IP/SOR / innym:

data..................................godz...........min..........

Stwierdzenie zgonu / odstąpienie od med. czynności rat.

data..................................godz...........min.........

41) Załącznik w brzmieniu ustalonym przez § 1 pkt 29 rozporządzenia, o którym mowa w odnośniku 3.

EMERGENCY MEDICAL SERVICE CARD

I HISTORY

LOCATION

Home
Public place
Traffic
Workplace
School
Farm

II EXAMINATION

GLASGOW COMA SCALE

<u>EYE OPENING</u>	
Spontaneously	4
To speech	3
To pain	2
No response	1
<u>VERBAL RESPONSE</u>	
Well oriented	5
Confused	4
Inappropriate words	3
Incomprehensible sounds	2
No response	1
<u>MOTOR RESPONSE</u>	
Obeys commands	6
Localises pain	5
Normal flexion withdrawal	4
Abnormal flexion	3
Abnormal extension	2
No response	1

SCORE:

RTS

RESPIRATORY RATE

10-29	**4 pts**
>29	**3 pts**
6-9	**2 pts**
1-5	**1 pts**
NONE	**0 pts**

SYSTOLIC BLOOD PRESSURE

>89	**4 pts**
76-89	**3 pts**
50-75	**2 pts**
1-49	**1 pts**
NONE	**0 pts**

GCS

13-15	**4 pts**
9-12	**3 pts**
6-8	**2 pts**
4-5	**1 pts**
3	**0 pts**

RTS SCORE:

RESPIRATORY SYSTEM

Respiratory rate (/min)

Signs and symptoms

Dyspnea	YES/NO
Cyanosis	YES/NO
Apnea	YES/NO

Auscultation (R and L side separately)

Vesicular sounds
Rhonchi
Weezing
Fine crackles
Coarse crackles
No sounds
Other

Oxygen saturation (%)

PUPILS

Light reflex (R and L side separately)

normal
slow
absent

SIZE

normal
myosis
mydriasis

BLOOD PRESSURE (mmHg)

PULSE (/min)

(regular/irregular)

Trauma – graphical representation (affected body part to be marked on a pictogram)

- none
- open fracture
- closed fracture
- sprain
- contusion
- wound
- bleeding
- crush
- amputation
- non-traumatic pain
- burn

Burn area and severity estimation (%)

Anatomical criteria for admission to Trauma Center:

- Penetrating injury to head and torso or blunt trauma with signs of injury of internal organs of the head, chest and abdomen
- Traumatic amputation above knee or elbow
- Extensive extremities crush injury
- Spinal cord injury
- Extremity fractures with signs of vascular or neural injury
- Fracture in two or more long bones and pelvic fracture

SIGNS

**- Shock**	YES/NO
**- Cardiac arrest**	YES/NO
**- Meningeal signs**	YES/NO
**- Convulsions**	YES/NO
**- Aphasia**	YES/NO
**- Vomiting**	YES/NO
**- Diarrhoea**	YES/NO
**- Bleeding**	YES/NO
**- Oedema**	YES/NO
**- Syncope**	YES/NO

OTHER

- Pregnancy
- Labour
- Infectious disease

SKIN

Appearence

- Normal
- Pale
- Erythema
- Jaundice
- Peripheral cyanosis
- Central cyanosis

Texture

- Normal
- Moist
- Dry

Temperature

- Normal
- Cold
- Warm

ABDOMEN

- Normal
- Tenderness to palpation
- No peristalsis
- Peritoneal signs

PSYCHOMOTOR ASSESMENT

- Normal
- Inhibited
- Agitated
- Aggressive

HEART SOUNDS

- Clear/loud
- Muffled
- Other

PARALYSIS

(side L and R separately)

- Upper extremity
- Lower extremity

BREATH SMELL

- Acetone
- Alcohol
- Other

GLUCOSE LEVEL (mg% or g/l)

ECG

- Sinus rythm
- Supraventricular tachycardia
- Ventricular tachycardia
- Atrial fibrillation
- Atrioventricular block
- Supraventricular extrasystoles
- Ventricular extrasystoles
- VF/VT
- Asystole
- PEA
- ICD
- Acute coronary syndrome
- Myocardial infarction
- Other

EXAMINATION – Open text

III – DIAGNOSIS (three ICD-10 codes)

IV – MEDICAL INTERVENTIONS

INTERVENTION

Suction
Manual ventilatory support
Oropharyngeal tube
Tracheal intubation
Mechanical ventilation
Oxygen supplementation
Defibrillation
External stimulation
Cardioversion
Chest compressions
Teletransmission
Collar
Spinal board
Vacuum mattress
Stabilisation
Dressing
Peripheral vein cannulation
Central vein cannulation
Urinary catheter placement

Gastric tube placement
Monitoring
Other

DRUGS AND EQUIPMENT USED (open text)

V - PERSONAL DATA AND PATIENT HANDOVER

PATIENT DATA
Name
Surname
Adress
ID number

NFZ code
Birth date/Age
Personal Identification Number (PESEL)

Signature and stamp of the emergency team leader

Date

Healthcare facility decision
Admission
Admission refusal

Emergency department stamp
ED doctor stamp

Patient handover to ED:
Date and time

Confirmation of death/abandonment of medical rescue
Date and time
